# Accumulation and Transformation of Biogenic Amines and Gamma-Aminobutyric Acid (GABA) in Chickpea Sourdough

**DOI:** 10.3390/foods10112840

**Published:** 2021-11-17

**Authors:** Tomaž Polak, Rok Mejaš, Polona Jamnik, Irena Kralj Cigić, Nataša Poklar Ulrih, Blaž Cigić

**Affiliations:** 1 Biotechnical Faculty, University of Ljubljana, Jamnikarjeva 101, SI-1000 Ljubljana, Slovenia; tomaz.polak@bf.uni-lj.si (T.P.); mejas.rok@gmail.com (R.M.); polona.jamnik@bf.uni-lj.si (P.J.); natasa.poklar@bf.uni-lj.si (N.P.U.); 2 Faculty of Chemistry and Chemical Technology, University of Ljubljana, Večna pot 113, SI-1000 Ljubljana, Slovenia; irena.kralj-cigic@fkkt.uni-lj.si

**Keywords:** biogenic amines, gamma-aminobutyric acid, chickpea sourdough, fermentation, diamine oxidases

## Abstract

In general, sourdough fermentation leads to an improvement in the technological, nutritional, and sensory properties of bakery products. The use of non-conventional flours with a specific autochthonous microbiota may lead to the formation of secondary metabolites, which may even have undesirable physiological and toxicological effects. Chickpea flours from different suppliers have been used to produce sourdoughs by spontaneous and inoculated fermentations. The content of nutritionally undesirable biogenic amines (BA) and beneficial gamma-aminobutyric acid (GABA) was determined by chromatography. Fenugreek sprouts, which are a rich source of amine oxidases, were used to reduce the BA content in the sourdoughs. Spontaneous fermentation resulted in a high accumulation of cadaverine, putrescine, and tyramine for certain flours. The use of commercial starter cultures was not effective in reducing the accumulation of BA in all sourdoughs. The addition of fenugreek sprouts to the suspension of sourdough with pH raised to 6.5 resulted in a significant reduction in BA contents. Enzymatic oxidation was less efficient during kneading. Baking resulted in only a partial degradation of BA and GABA in the crust and not in the crumb. Therefore, it could be suggested to give more importance to the control of sourdough fermentation with regard to the formation of nutritionally undesirable BA and to exploit the possibilities of their degradation.

## 1. Introduction

Sourdough is becoming increasingly popular and plays an important role in bread making as it improves the properties of dough and bread. In traditional production, the mixture of flour and water is fermented spontaneously [1]. It is mostly done by experience, and the raw materials and technologies are not standardized [2]. In addition, the microbial contamination of the flour, baking equipment, and even the microbiota on the baker’s hands [3] contribute to a greater diversity of fermentation. Both yeasts and lactic acid bacteria (LAB) have been isolated from spontaneously fermented doughs, but generally, LAB are the dominant microorganisms [4]. The process is less controlled than when breads are made with yeasts *Saccharomyces cerevisiae*, which are routinely used as leavening agents. However, consumers perceive sourdough bread as healthier and less “industrial” and are therefore willing to pay more for such breads [5,6]. The use of sourdough instead of yeast as a leavening agent has many advantages from a sensory, technological, and nutritional point of view [7]. The complex microbiota in sourdough results in complex flavors from various acids, alcohols, esters, carbonyls, phenols, amines, and sulfur-containing compounds [8]. Partial degradation of large starch molecules is beneficial in regard to stalling [9], which together with the antimicrobial and antifungal activity of lactic and acetic acids, contributes to the longer shelf life of bread. The better digestibility of proteins and lower antigenicity, formation of bioactive peptides, release and synthesis of antioxidants, partial degradation of phytate, and better accessibility of minerals are some of the nutritional benefits attributed to sourdough fermentation [4,10].

Traditionally, whole rye or wheat flour is used for the preparation of sourdough [11], although various pseudocereals and legumes [12] have also been found to be efficient substrates for fermentation. Chickpea is a legume whose global production has increased significantly in recent years [13] and can be used in various salads, stews, or in fried form as falafel [12]. Chickpea flour is a staple food in the Indian subcontinent [14] and is also one of the most popular legume flours in the Western world [15]. In recent years, fermented chickpea flour has been used in various foods such as pasta [16], bakery products [17], bread [18,19,20,21], and gluten-free bread [22]. Not only new but also traditional uses of fermented chickpea have been recognized. Artakena, the traditional bread from Cyprus made from spontaneously fermented chickpea flour [23], has been included in the Intangible Cultural Heritage section of UNESCO.

Although several beneficial effects have been observed, less attention has been paid to the safety of such fermented foods, particularly in relation to the formation of biogenic amines (BA). BAs are present in several fermented foods, and LAB are considered to be the main producers of BA including the following genera *Enterococcus*, *Lactobacillus*, *Streptococcus*, *Lactococcus Oenococcus*, *Pediococcus*, *Weissella*, *Carnobacterium*, *Tetragenococcus*, *Leuconostoc*, and *Sporolactobacillus* [24] BA such as tyramine (TYR), cadaverine (CAD), and putrescine (PUT) can reach levels above 100 mg/kg in various cheeses, fermented vegetables such as cabbage and turnip, sausages, and especially fermented soy products [25]. Reports of biogenic amine formation in sourdough are relatively rare, which is unusual given the widespread use and increasing popularity of sourdough, which is often produced under low controlled spontaneous fermentation. However, some reports indicate the potential problem, as LAB with great potential to form BAs have already been isolated from sourdough [26,27,28], and BA can accumulate in the fermented soybeans [29] and lupin flour [30].

Legumes and especially their sprouts are rich sources of diamine oxidases, which are the enzymes that catalyze the oxidation of polyamines and some monoamines to their corresponding aldehydes [31]. Diamine oxidases of animal origin ingested in capsules were efficient in oxidizing unwanted dietary biogenic amines [32]. The problem with such application is that the reaction produces H_2_O_2_, which is toxic to intestinal cells. Alternatively, the oxidation of biogenic amines can be done in the food matrix itself before consumption. Fenugreek sprouts, which are a rich source of diamine oxidases [33], can potentially be used to reduce the undesirable BA content in sourdoughs.

Spontaneously fermented chickpea flour is one of the ingredients of a traditional fermented cereal food from Turkey called Kumru [34], in which biogenic amines were found at a total concentration of less than 4 mg/100 g of the product. TYR (42 mg/kg) was also found in experimental bread prepared from wheat flour sourdough using *Levilactobacillus brevis* CECT 8183, with a high potential for the production of gamma-aminobutyric acid (GABA), as a starter [35]. The high content of GABA in food, which is mainly formed by the decarboxylation of glutamate [36], is highly desirable in contrast to BAs [24].

The aim of the present study is to evaluate the difference in the profiles of BA and GABA in spontaneously fermented (I) and inoculated (II) chickpea flour of different origins, to evaluate the enzymatic potential of diamine oxidases from sprouts to degrade undesirable BA in sourdough and dough before baking (III) and (IV) to assess the influence of baking on the content of BA in bread.

## 2. Materials and Methods

### 2.1. Materials

Acetonitrile (gradient HPLC grade) was obtained from Fischer Scientific (Hampton, NH, USA). Ultrapure water was obtained with a Milli-Q water system (Millipore Merck, Darmstadt, Germany). Acetone (≥99.8%), n-hexane (≥95%), and HCl (37%) were obtained from Honeywell (Charlotte, NC, USA), NaOH (p.a.); NH_3_ (25%), acetic acid (glacial), NaH_2_PO_4_ × 2H_2_O (p.a.), and NaHCO_3_ (p.a.) were obtained from Merck (Darmstadt, Germany). Trolox, Folin–Ciocalteu reagent, dansyl chloride (≥99%), GABA (≥99%), and amines: 1,7-diaminoheptane (98%), phenethylamine (99%), histamine (≥97%), cadaverine (≥96.5%), putrescine (≥98.5%), spermidine (≥98%), spermine (≥97%), tyramine (≥98.5%), and tryptamine (≥98%) were obtained from Sigma-Aldrich (St. Louis, MO, USA).

Three wholemeal chickpea flours (Flour-1, Flour-2, Flour-3) with a certificate of organic origin from different suppliers were obtained in specialized shops for organic products in Ljubljana, Slovenia. All experiments with flours were performed prior to the best-before date marked on the packaging. Commercial starter culture Livendo^TM^ starter LV1 was obtained from Lesaffre (Lille, France).

### 2.2. Spontaneous and Inoculated Fermentation of the Chickpea Flours

For spontaneous fermentation, 5 g of each flour and Milli-Q water (10 mL) were homogenized with a sterile glass rod in a 50 mL sterile polypropylene centrifuge tube. Fermentation locks were placed on the centrifuge tubes, and the suspensions were transferred to the incubator at a temperature of 30 °C. For each flour, two tubes were prepared for fermentation. One tube was incubated for 24 h, and the other was incubated for 48 h. Immediately after fermentation, the airlocks were removed, and the tubes containing the sourdoughs were sealed and stored at −20 °C for a maximum of one week until the extraction of BAs (Section 2.3.1) and antioxidants (Section 2.4) was performed. For each flour, three independent fermentations were performed within three consecutive weeks.

For inoculated fermentation, a suspension of flour and Milli-Q water was prepared in a polypropylene centrifuge tube in the same way as for spontaneous fermentation. Before attaching a fermentation lock, a lyophilized commercial starter culture (LivendoTM Starter LV1) was added according to the manufacturer’s instructions (mass ratio of starter to flour 1:200), and the suspension was thoroughly mixed with a glass rod. The latter protocols were the same as for spontaneous fermentation.

### 2.3. Determination of BAs and GABA by HPLC

#### 2.3.1. Extraction Procedure

Approximately 1.0 g (the exact mass was known) of the sourdough suspension was weighed into 15 mL polypropylene centrifuge tubes, which was followed by the addition of 10 mL of 0.4 M HCl containing 10 mg/L 1,7-diaminoheptane as an internal standard (IS). The contents were mixed thoroughly with a laboratory vortex (30 s) and incubated at 25 °C for 30 min. After 10 min and 20 min of incubation, additional mixing was performed (30 s). After extraction, an aliquot of 1.5 mL was transferred to 2 mL centrifuge tubes and centrifuged at 10,000× *g* for 5 min. The clear supernatant was used for derivatization.

#### 2.3.2. Derivatization with Dansyl Chloride (DNS–Cl)

Derivatization was performed in a 1.5 mL centrifuge tube, as previously described [33]. First, 250 μL of calibration solution (mixture of standards in 0.4 M HCl) or sample (clear supernatant) was pipetted, and then, 50 μL of 2 M NaOH, 75 μL of saturated solution of NaHCO_3_ and 500 μL of DNS–Cl solution (10 g/L in acetone) were added with each addition mixed with a vortex. Derivatization was performed in a heating block at 40 °C for 60 min. After incubation, 25 μL of a 25% aqueous NH_3_ solution was added and allowed to stand at room temperature for 30 min. Subsequently, 350 μL of acetone was added, and the solution was mixed again. The supernatant was filtered through a 0.45 μm nylon filter before HPLC analysis.

#### 2.3.3. HPLC Analyses

The dansylated derivatives were separated on a Kinetex XB-C18 (5 μm, 100 Å, 150 × 4.6 mm) column with a guard column of the same particle size (Phenomenex, Torrence, CA, USA) on Agilent HPLC system 1100 (Palo Alto, CA, USA), as described previously [33]. The conditions used were as follows: column temperature, 30 °C; injection volume, 10 µL; and mobile phase flow rate, 700 µL/min. The components of the mobile phase were Milli-Q water (eluent A) and acetonitrile (eluent B). The mobile phase gradient was programmed as follows (% B): 0–25 min, 40–80%; 25–30 min, 80–100%; 30–35 min, 100%; 35–40 min, 100–40%; 40–42 min, 40%. The wavelength of the UV-vis detector was 254 nm, the excitation wavelength of the fluorescence detector was 350 nm, and the emission wavelength was 520 nm. Due to the better sensitivity and selectivity of the dansylated amines obtained with a fluorescence detector, the signals for the latter were used for peak area integration and further evaluation. The only exceptions were HIS and GABA, for which the spectrophotometric signals were used because the fluorescence yield of the dansylated derivatives was low. Slopes of calibration curves for biogenic amines and GABA obtained by both detectors are shown in Table A1 (Appendix A). All peak areas were normalized to those of IS, which was used in amine standard solutions and included in the extraction buffer for sample preparation to control all steps of sample manipulation from extraction, derivatization, and injection into HPLC. The matrix showed only a small influence on the derivatization yield of IS. The median derivatization yield of IS in the complex matrix was 84% (upper quartile 89% and lower quartile 79%).

### 2.4. Folin–Ciocalteu Assays of Antioxidants

The free phenolic compounds and other antioxidants in the sourdough suspensions were extracted using 80% (*v*/*v*) acetone in Milli-Q water, with continuous mixing in a water bath at 40 °C for 20 min [37]. The contents of the soluble antioxidants obtained after centrifugation (4000× *g*, 10 min) were determined using Folin–Ciocalteu assays. The antioxidant potentials are expressed as Trolox equivalent (TE) antioxidant capacity [38].

### 2.5. Enzymatic Degradation of BA

Fenugreek sprouts, which are a rich source of amine oxidases, were grown and harvested as previously described [33]. Fresh sprouts were lyophilized and homogenized using a ball homogenizer (MM40, Retsch, Haan, Germany) for 30 s with a frequency of 30 s^−1^, stored at room temperature, and used for experiments within one week after homogenization.

#### 2.5.1. Enzymatic Degradation of BA in the Sourdough Suspension

Sourdough was prepared by the spontaneous fermentation of Flour-2 for 48 h as described in Section 2.2. After fermentation, 4 g aliquots of sourdough were weighed into two 100 mL glass beakers. Then, 16 mL of Milli-Q water was added to one beaker. The pH of the sourdough suspension was 4.7. The pH of the sourdough in the other beaker was raised to 6.5 by adding 4 M NaHCO_3_, and the volume up to 16 mL was balanced by Milli-Q water. The contents of both beakers were continuously stirred at 25 °C with a magnetic stirrer at 500 rpm. To each beaker, 0.56 g of lyophilized sprouts were added, and aliquots of 0.75 mL were immediately pipetted (10 s after addition of sprouts) into a 2 mL microcentrifuge tube containing 0.9 mL of 0.67 M HCl. The BAs in these solutions were determined and represented time zero (100% of the respective BA). Sampling (aliquots of 0.75 mL) was repeated after 3, 15, 45, and 90 min of stirring, and the determined content of BA in these solutions was used to evaluate the efficiency of BAs oxidation by diamine oxidases.

#### 2.5.2. Enzymatic Degradation of BA in the Dough

The model dough was prepared from 300 g of white wheat flour type 400, 5.9 g of salt, and 2.3 g of dry yeast, which was weighed in a bowl. To this dry mass, 200 mL of standard solutions of Bas—PUT, CAD, and TYR each with a concentration of 140 mg/L—were added and kneaded for 3 min with the kitchen robot to obtain a homogeneous mass. Then, 50 mL of sprouts suspension (3 g of lyophilized and homogenized fenugreek sprouts suspended in 50 mL of Milli-Q water and stirred for 5 min with a magnetic stirrer) was added to the mass, and samples were taken (protocol as in Section 2.3.1) after 3 min, 8 min, 10 min, and 15 min of continuous kneading. Then, we let the dough rest for another 45 min. Two samples were also taken 30 min and 60 min after the addition of sprouts suspension.

### 2.6. Influence of Baking on the Content of BA in Bread Prepared from Sourdough

The sourdough used for this experiment was prepared from Flour-2 using spontaneous fermentation at 30 °C for 24 h (large scale—120 g flour and 240 mL Milli-Q water). The dough was prepared from 150 g of the previously prepared sourdough, 250 g of white wheat flour type 400, 5.9 g of salt, 2.3 g of dry yeast, 150 mL of Milli-Q water, and 1.8 g of NaHCO_3_ (to increase the pH of the dough to 5.9). The entire mixture was kneaded for 15 min using the kitchen robot (Heavy Duty 4.8 L KitchenAid, Whirlpool, Benton Charter Township, MI, USA). After kneading, the dough was allowed to rise for an additional 45 min. Three independent samples were taken from different parts of the dough, and the BAs and GABA were extracted as in Section 2.3.1, with an additional homogenization procedure using T-25 Ultra-Turrax (Ika-Labortechnik, Staufen, Germany) at 13,500 rpm (30 s homogenization/30 s rest period—repeated 3 times). The dough was placed in the oven and baked at 200 °C for 45 min. After the bread had cooled, three independent samples of the bread crust and bread crumb were taken and used for extraction with the same protocol as for the dough in this section.

### 2.7. Measurement of pH and Determination of Dry Weight

The pH values (Table A2 in Appendix A) were measured directly using a combined glass–gel spear electrode (type 03, Testo pH electrode) with a thermometer (type T, Testo penetration temperature probe) connected to a pH meter (Testo 230, Testo, Titisee-Neustadt, Germany).

The dry weight of all samples was determined by oven drying the samples at 105 °C to constant mass (≈6 h).

### 2.8. Statistical Analysis

A non-parametric Mann–Whitney test [39,40] based on the data ranking was used for the statistical analysis. The differences in the content of a particular polyamine in samples were significant at the *p* < 0.05 level.

## 3. Results and Discussion

### 3.1. Formation of Nutritionally Undesirable Biogenic Amines in the Sourdoughs of the Chickpea Flours 

**Figure 1:**
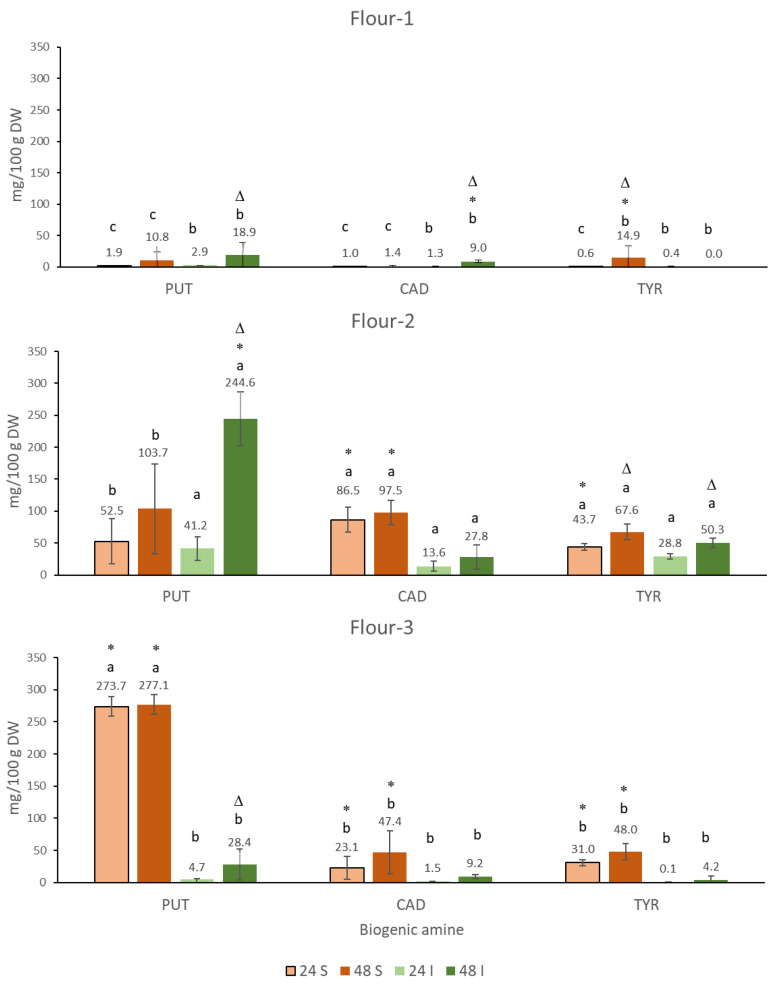
Putrescine (PUT), cadaverine (CAD), and tyramine (TYR) in chickpea sourdoughs from Flour-1, Flour-2, and Flour-3 prepared by spontaneous fermentation (S) and inoculated fermentation with commercially available starter culture (I) after 24 h and 48 h of fermentation at 30 °C. The differences in the content of particular compound that are attributed to flour origin at fixed duration and type of fermentation are marked with different letters (a, b, c); different duration of fermentation at fixed flour origin and type of fermentation is marked with Δ; type of fermentation at fixed flour origin and duration of fermentation is marked with *. The differences were significant at the *p* < 0.05 level.

The BAs PUT, CAD, and TYR typically accumulate the most in fermented foods of plant origin [41]. Fermentation of the three organic chickpea flours resulted in the accumulation of these three BAs. The origin (supplier) of the flour, the fermentation time, and the addition of a commercial starter culture have a selective influence on the accumulation of a particular biogenic amine. None of the BAs analyzed accumulated to the higher levels after 24 h compared to 48 h, when the same type of fermentation (spontaneous or inoculated) was used. Significantly higher contents after longer incubation were found only for certain BAs and flours, depending on the variability of the determined contents in the respective samples (Figure 1). The addition of the starter culture was relatively efficient in reducing BA content in two flours, while the fermentation of one flour resulted in only a partial reduction in the content of CAD and TRM and even in a significant increase in PUT to levels above 200 mg/100 g DW.

#### 3.1.1. Putrescine (PUT)

The highest values were found for PUT (Figure 1) after 24 h and 48 h of spontaneous fermentation of Flour-3 (270 mg/100 g DW). These values are among the highest found in all fermented foods, including those of plant or animal origin [24]. In the case of spontaneous fermentation of Flour-2, the values were several times lower. The large relative standard deviations for the PUT content in Flour-2 at both time points indicate that spontaneous fermentation was not reproducible. Spontaneously fermented Flour-1 is characterized by a much lower PUT content, as only 1.9 mg/100 g DW was accumulated after 24 h, which is more than 100 times lower than the content in Flour-3. The variation of PUT content in certain spontaneously fermented foods such as traditional cheeses [42] or vegetable matrices [24] can be very high. Controlled fermentation, achieved by a combination of pasteurization, addition of starter cultures, and higher salt content generally results in a lower accumulation of all biogenic amines [43].

The addition of commercial sourdough starter cultures as recommended by the supplier resulted in a large and significant reduction in PUT content in Flour-3, as 2 orders of magnitude less PUT content was detected after 24 h and after 48 h of incubation. The differences could be due to the selection of microbiota with low potential to convert PUT from arginine or alternatively with high diamine oxidase activity that would lead to PUT oxidation. In contrast to Flour-3, the addition of the starter to Flour-2 actually resulted in a significant increase in PUT content after 48 h to levels that were an order of magnitude higher than in Flour-3 fermented with the starter. Thus, the addition of starter cultures may actually contribute to a higher accumulation of nutritionally undesirable PUT. In Flour-1, the content of PUT was not affected by the addition of starter cultures. 

#### 3.1.2. Cadaverine (CAD)

**Figure A1:**
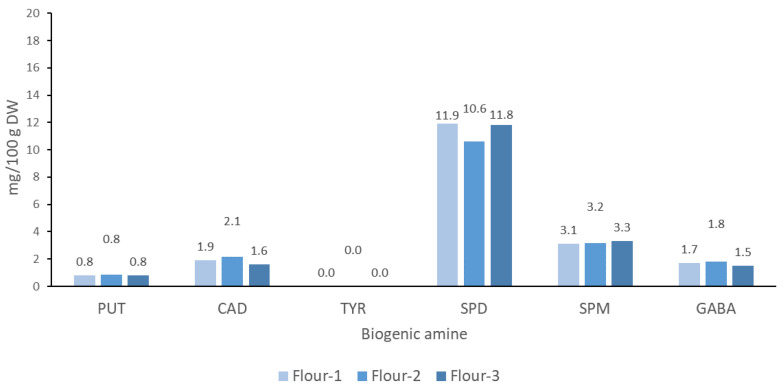
Putrescine (PUT), cadaverine (CAD), tyramine (TYR), spermidine (SPD), spermine (SPM) and gamma-aminobutyric acid (GABA) in chickpea flours: Flour-1; Flour-2, Flour-3.

Spontaneous fermentation resulted in the accumulation of CAD in two of the three chickpea flours (Figure 1). The highest levels of CAD were found in the sourdough of Flour-2 both after 24 h (86.5 mg/100 g) and after 48 h (97.5 mg/100 g) of fermentation, where CAD accumulated in a similar range as PUT. Such high levels of CAD are rarely found in foods. Among fermented foods, only certain acid-cured cheeses [44] and soy sauces [45] contain more CAD on a DW basis. The exception with high CAD contents is also some legume sprouts, but in these, CAD accumulates due to endogenous biosynthesis rather than due to microbial decarboxylases [33]. The contents of CAD in the sourdough of Flour-3 (47.4 mg/100 g; 48 h) were significantly higher than in spontaneously fermented Flour-1 (1.4 mg/100 g, 48 h) and lower than in Flour-2 after both incubation periods. The CAD content in the sourdough of Flour-1 (Figure 1) was not increased compared to the unfermented flour (Figure A1 in Appendix A), suggesting that the lysine decarboxylase activity of the microbial population was relatively low or, alternatively, the diamine oxidase activity was relatively high, which would also explain the low PUT accumulation in spontaneously fermented Flour-1. Significant differences in the ratio and absolute content of PUT and CAD in each of the spontaneously fermented chickpea flours indicate the wide diversity of the microbiota and the course of fermentation in the chickpea flours of different origins. 

Inoculation with a commercial starter cultures resulted in significantly lower CAD contents in the sourdoughs of Flour-2 and Flour-3 compared to the spontaneously fermented flours, indicating the efficiency of the starter cultures in reducing CAD contents. 

CAD and PUT are polyamines that bind to human trace amine-associated receptors [46], which generally prevents us from consuming the decomposed foods. CAD is also slightly more toxic to intestinal cells than PUT [47]; it potentiates the toxicity of HIS and TYR and contributes to the formation of nitrosamines [48]. CAD and PUT contribute to some extent to the typical flavor of certain foods [49], but their presence in foods is generally undesirable.

#### 3.1.3. Tyramine (TYR)

The highest TYR contents were found for Flour-2 (67.6 mg/100 g) and Flour-3 (48.0 mg/100 g) after 48 h of fermentation (Figure 1). Not much lower levels were found after 24 h of incubation, indicating the problem of TYR accumulation in spontaneously fermented chickpea flour, which is actually used in typical food products [23]. Even in Flour-1 (14.9 mg/100 g), where the accumulation of biogenic amines was least pronounced, the TYR content was statistically indistinguishable from Flour-3 after 48 h of spontaneous fermentation.

Fermentation with a starter culture resulted in a statistically significant lower content of TYR in Flour-3 after 48 h (4.2 mg/100 g) compared to spontaneous fermentation and TYR contents below the limit of detection (<0.1 mg/100 g) in all fermentation batches of Flour-1. Starter was less efficient in reducing TYR content in Flour-2, as the contents after 48 h (50.3 mg/100 g) were statistically not different in comparison to spontaneous fermentation. TYR contents of similar magnitude in the sourdoughs of Flour-2 were previously found in some samples of sauerkraut or acid cured cheeses [50]. Next to HIS, TYR is the least desirable biogenic amine in food. Its toxicity to intestinal cells is even greater than that of HIS [51], which, together with the effect of TYR on higher blood pressure after ingestion, may lead to an acute health crisis in susceptible individuals [52]. EFSA stated that the intake of TYR within a meal should not exceed 50 mg in individuals taking third-generation monoamine oxidase inhibitors (MAOIs) drugs and as much as 6 mg in individuals taking classical MAOI drugs [53]. Such amounts could be easily exceeded by the consumption of bread made from Flour-2 sourdough both in the presence and absence of the starter.

#### 3.1.4. Tryptamine (TRM), Phenethylamine (PEA), and Histamine (HIS)

**Figure A2:**
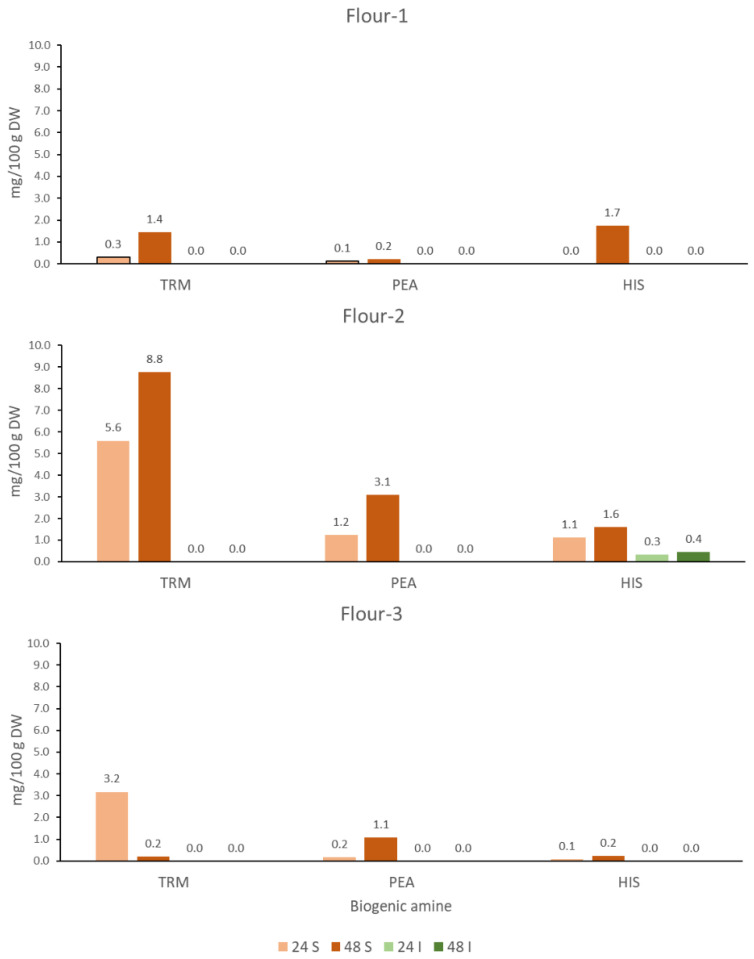
Tryptamine (TRM), phenethylamine (PEA) and histamine (HIS) in chickpea sourdoughs from Flour-1; Flour-2, Flour-3, prepared by spontaneous fermentation (S) and inoculated fermentation with commercially available starter culture (I) after 24 h and 48 h of fermentation at 30 °C.

The biogenic amines TRM, PEA, and HIS accumulated in much lower amounts than PUT, CAD, and TRM, and the average levels in neither sourdough exceeded 10 mg/100 g (Figure A2 in Appendix A). The highest average level (8.8 mg/100 g) was found for TRM in spontaneously fermented Flour-2 (48 h). Even here, TRM was found only in three of five batches. The addition of starter was very efficient in reducing TRM content, as it was not found in any batch after 24 h and 48 h fermentation of all three flours. The nutritionally problematic HIS was determined in some batches of the flours fermented spontaneously or with the starter culture, yet the highest average content was only 1.8 mg/100 g and was determined after 48 h of spontaneously fermented Flour-1, and in none of the individual batches (48 in total) was the HIS content above 5.5 mg/100 g and therefore not found to be problematic BA in fermented chickpea flour. These results are consistent with literature data that HIS is mainly problematic in fermented/spoiled fish [54] and much less so in fermented foods of plant origin [41]. PHE was not found in any batch of the three flours fermented with starter cultures. Even in spontaneous fermentation, the average level did not exceed 3.1 mg/100 g (Flour-2, 48 h), and in none of the batches did it exceed 5.6 mg/100 g (Flour-2, 48 h).

### 3.2. Nutritionally Desirable BAs and GABA in the Sourdoughs of the Chickpea Flours 

#### 3.2.1. Spermidine (SPD) and Spermine (SPM)

Various legumes are nutritionally important sources of polyamines SPD and SPM [55,56]. High dietary intake of spermidine has been shown to be particularly beneficial in terms of prolonging human life span [57]. The SPD (10.8–11.9 mg/100 g) and SPM (3.1–3.3 mg/100 g) contents of unfermented flours are within the range of the previously reported data on the content in various legumes [58]. The determined SPD content for canned chickpeas is in the range of 8 mg/100 g DW and of SPM 0.5 mg/100 g. This is less than we found for the chickpea flours, which is probably due to the partial dissolution of both polyamines in aqueous media due to their high polarity [56].

In contrast to the nutritionally undesirable polyamines that accumulate during fermentation (Figure 1, Figure A2 in Appendix A), the levels of SPM and SPD after fermentation were not statistically different from those in flour. These results are consistent with previous findings that the levels of SPD and SPM are often not increased after fermentation [59]. In fact, the induction of degradation and expression of enzymes catalyzing the conversion of SPM and SPM during bacterial degradation was found [60]. In order to increase the SPM and SPD content, the application of specific cultures is required [61,62]. 

#### 3.2.2. Gamma-Aminobutyric Acid (GABA)

**Figure 2:**
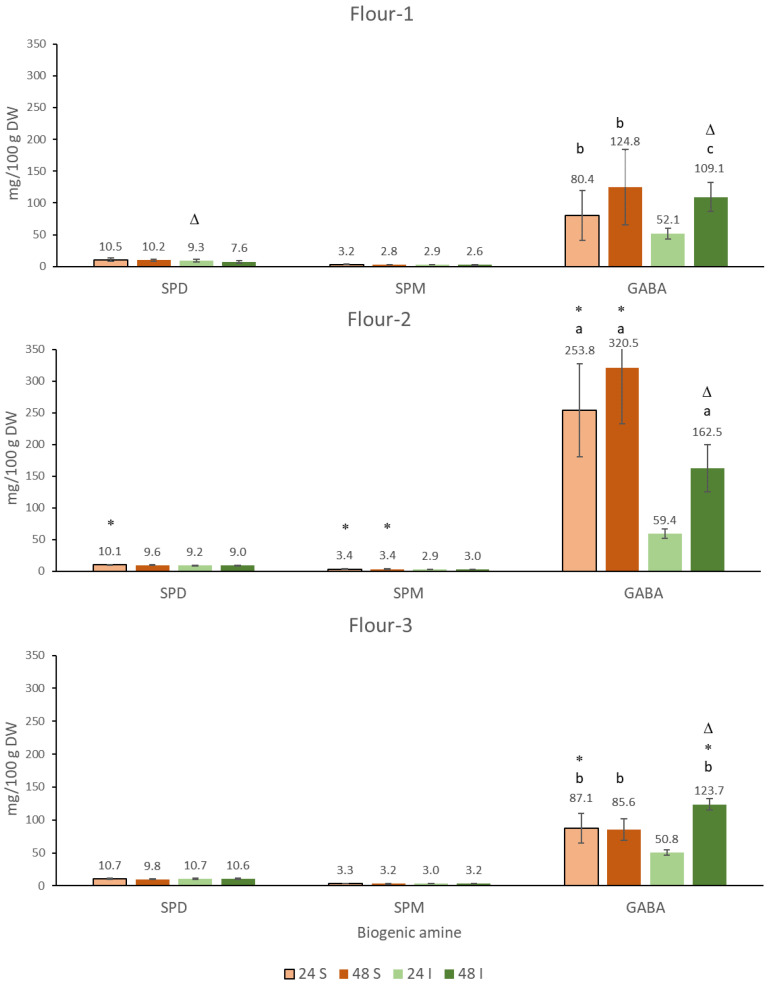
Spermidine (SPD), spermine (SPM), and gamma-aminobutyric acid (GABA) in chickpea sourdoughs from Flour-1, Flour-2, and Flour-3 prepared by spontaneous fermentation (S) and inoculated fermentation with commercially available starter culture (I) after 24 h and 48 h of fermentation at 30 °C. Differences in the content of particular compounds that are attributed to flour origin at fixed duration and type of fermentation are marked with different letters (a, b, c); different duration of fermentation at fixed flour origin and type of fermentation is marked with Δ; type of fermentation at fixed flour origin and duration of fermentation is marked with *. The differences were significant at the *p* < 0.05 level.

Fermentation of all three flours resulted in a strong accumulation of GABA (Figure 2). The highest content was found in spontaneously fermented Flour-2, 253.8 mg/100 g after 24 h and 320.5 mg/100 g after 48 h, but the values were not statistically different. Such high levels are rarely found in fermented flours, even when starters with high potential for GABA accumulation are applied [63]. The spontaneous fermentation of Flour-1 and Flour-3 resulted in significantly lower GABA accumulation, ranging from 80.4 to 124.8 mg/100 g depending on the flour and fermentation time, although the samples were not statistically different. The sourdoughs from Flour-2 with the highest content of nutritionally undesirable BAs CAD and TYR were also those with the highest GABA content. The addition of starter to Flour-2 resulted in significantly lower GABA content, as 4.3 times less GABA was determined after 24 h and 2.0 times less GABA was determined after 48 h than without starter. Considering that the addition of starter to Flour-2 did not result in a statistically significant decrease in TRM and even in a more than two-fold increase in PUT at 48 h (Figure 1), the addition of starter could be considered nutritionally undesirable. For Flour-1, the addition of starter can be considered neutral, as the content of BAs was low both in the presence and absence of starter, and the GABA content was also statistically indistinguishable (all values ranged from 52.1 to 124.8 mg/100 g). Only for Flour-3, the addition of starter was beneficial as more GABA was determined after 48 h with the starter (123.7 mg/100 g) than with spontaneous fermentation (85.6 mg/100 g). The addition of the starter to Flour-3 resulted in a significant reduction in the contents of PUT, CAD, and TYR and was therefore also beneficial in terms of a reduced accumulation of nutritionally undesirable BA. Considering the large reduction in the content of PUT upon the addition of starter to Flour-3 (Figure 1), it is possible that at least part of the GABA was not formed by the decarboxylation of glutamate, but from putrescine by the simultaneous action of microbial diamine oxidases and 4-aminobutiraldehyde dehydrogenases [36].

The formation of GABA is related to the same adaptive mechanism of microorganisms in which decarboxylation leads to an increase in pH. Since GABA is mainly formed by the decarboxylation of glutamate, the product is not BA but an amino acid [64]. Unlike BAs, which are formed by decarboxylation, GABA is nutritionally desirable, and various approaches using selected MO even in combination with germination [65] have been shown to be efficient in increasing GABA content. The use of chickpea sourdough for the preparation of experimental bread resulted in significantly higher GABA content in the final product in compared to commercial artisan breads, which were presumably prepared with sourdough starters based on cereal flour [35]. GABA-enriched bread obtained by the fermentation of legumes was also presented as a functional food [12].

### 3.3. Antioxidant Content of the Sourdoughs of the Chickpea Flours

**Figure 3:**
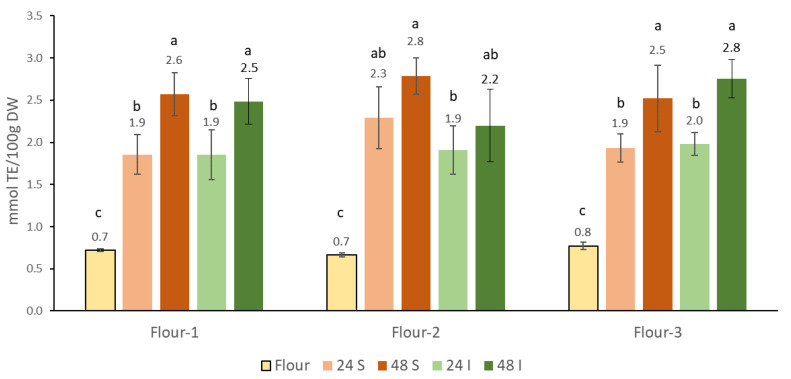
Influence of fermentation on the accumulation of antioxidants in three chickpea sourdoughs from Flour-1, Flour-2, and Flour-3 prepared by spontaneous fermentation (S) and inoculated fermentation with commercially available starter (I) after 24 h and 48 h of fermentation at 30 °C. Values marked with a different letter (a, b, c) differ significantly in antioxidant content for a particular flour and sourdoughs prepared from that flour. The differences were significant at the *p* < 0.05 level.

Chickpea flour is a relatively rich source of antioxidants, and catechin in the range of 1.5–2.0 mg/kg is the predominant polyphenolic compound [44]. The content of total antioxidants in three flours determined in the current study was in the range of 0.7–0.8 mmol TE/100 g, which is more than that determined in two other studies for chickpea seeds [44,66], with values in the range of 0.3 mmol TE/100 g and considerably less than 6 mmol TE/100 g, as determined in another study [67]. Many factors such as cultivars, agronomic practice, type of extraction, and even methods used to calculate and normalize the data [38] may contribute to the observed differences. All fermentations (Figure 3) resulted in significantly increased antioxidant content, ranging from 2.4-fold to 4.0-fold. A similar relative increase due to fermentation has been observed previously, and only 5% replacement of white flour with fermented chickpea flour resulted in approximately 20% higher antioxidant content in bread [20]. The addition of cultures did not affect the antioxidant content, as there were no differences for a given flour compared to spontaneous fermentation after 24 h or 48 h (Figure 3). Longer incubation times, on the other hand, resulted in higher antioxidant content, which was statistically significant only for sourdoughs made from Flour-1 and Flour-3. Microbial hydrolysis reaction or structural degradation of the cell wall are the most important factors contributing to higher antioxidant content after fermentation [68]. In addition to phenolic compounds, some other redox active compounds may also contribute to the higher values determined [69]. Since most of the polyphenols in chickpea are in bound form [66], release by endogenous or microbial hydrolases most probably contributes to an increase in the antioxidant content.

### 3.4. Application of Lyophilized Fenugreek Sprouts for the Reduction of Nutritionally Undesirable Biogenic Amines in the Sourdoughs and Bread

**Figure 4:**
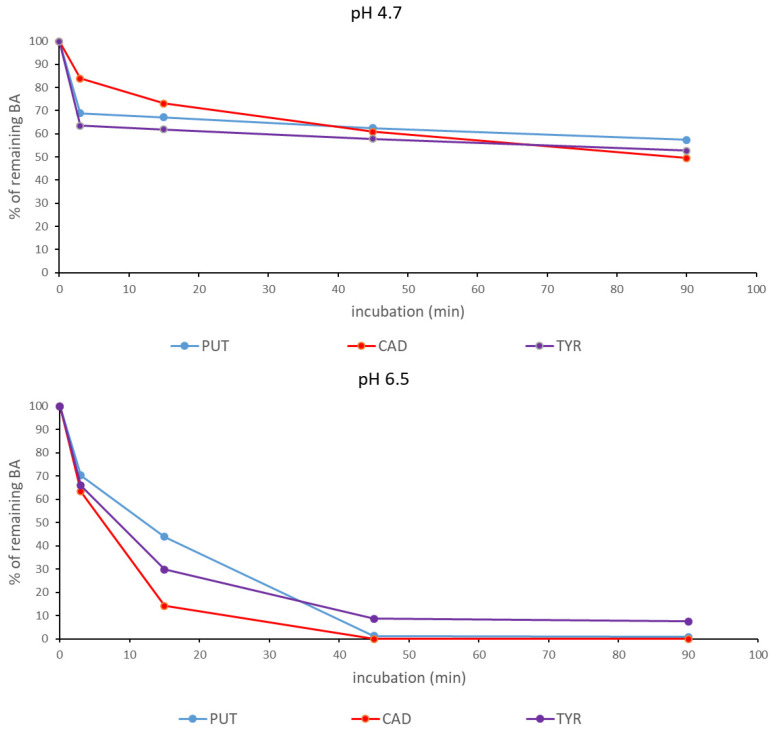
Degradation of biogenic amines (PUT—putrescine; CAD—cadaverine; TYR—tyramine) in the suspension of sourdough by diamine oxidase from fenugreek sprouts at pH 4.7 and pH 6.5.

We have previously shown that fresh fenugreek sprouts can be used to degrade BAs (PUT, CAD, TYR) in chemically defined systems [33]. As mentioned earlier in the manuscript, these polyamines may pose a potential health hazard to sensitive individuals. The consumption of capsules containing diamine oxidase can effectively reduce the level of polyamines and decrease the severity of migraine [32]. However, the enzymatic oxidation of BA in the digestive tract leads to the formation of H_2_O_2_, which is toxic to intestinal cells [70]. The oxidation of BA before food consumption could be an alternative. We tested whether lyophilized fenugreek sprouts could be used to reduce the content of BA in the suspension of chickpea sourdough (28 mg sprouts/g suspension) at a sourdough pH (4.7) and when the pH was increased to 6.5 (Figure 4). Lyophilized sprouts were partially efficient at reducing the BA content of the sourdough at a pH of 4.7 to less than 60% of the original content, while at pH 6.5 after 45 min, practically all PUT and CAD were oxidized, and TYR was reduced to less than 10% of the initial content. At a higher pH, more than 30% of BA was degraded after only 3 min of incubation. The lower efficiency at pH 4.7 could be due to both the lower activity and stability of diamine oxidase from legumes [71] compared to pH 6.5.

**Figure 5:**
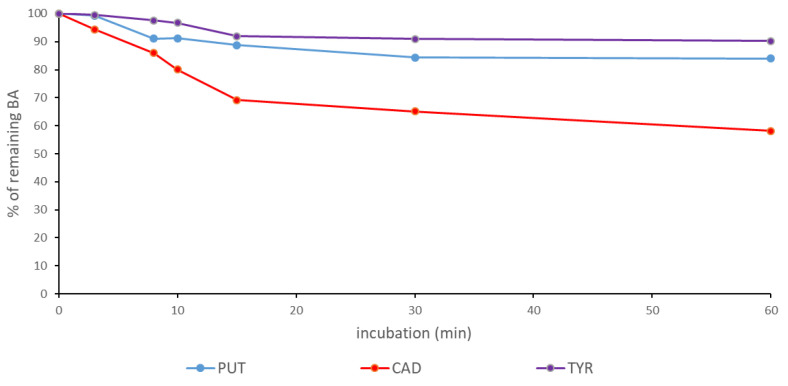
Degradation of biogenic amines (BA) (putrescine—PUT; cadaverine—CAD; tyramine—TYR) by diamine oxidase from fenugreek sprouts in the dough during kneading (0–15 min) and resting (15–60 min).

To test the efficacy in a real dough matrix, PUT, CAD, and TYR were incorporated in the dough at 25 mg/kg each along with lyophilized fenugreek sprouts (5.5 mg sprouts/g dough). The content of BA was determined within 15 min of kneading the dough and within a resting period of 45 min (Figure 5). The content of all three BA was reduced but to a lesser extent than in the suspension. After 60 min, the content of CAD was reduced by 42%, the content of PUT was reduced by 16%, and the content of TYR was reduced by 10%, which was mainly within the 15 min kneading phase. The reason for the lower efficiency of degradation could be the lower concentration of diamine oxidases (five-fold lower content of sprouts) and of BA than in suspension of sourdough. In addition, the irreversible inhibition of diamine oxidases by the radical intermediates that accumulate under low-oxygen conditions [72] could also contribute to a faster inactivation of the enzymes in the dough, where the diffusion of the gasses is much slower than in the stirred diluted suspension of the sourdough. All in all, fenugreek sprouts can be efficiently used to reduce the content of PUT, CAD, and TYR not only in model systems [33] but also in real food matrices, preferably in the sourdough suspension that can be later added to the flour. However, for a possible application of fenugreek sprouts in the context of reducing the content of BA, both the influence on sensory properties and health effects should be evaluated.

### 3.5. Influence of Baking on the Stability of Biogenic Amines in Bread

**Figure 6:**
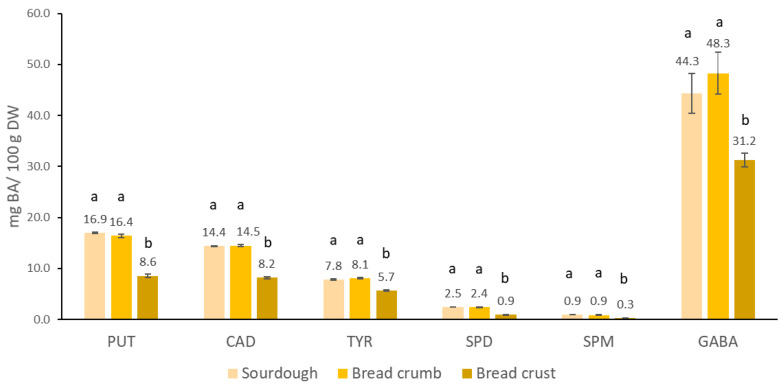
The content of biogenic amines (PUT—putrescine; CAD—cadaverine; TYR—tyramine; SPD—spermidine; SPM—spermine) and GABA in bread prepared from Flour-2 chickpea sourdough. Values marked with a different letter (a, b) differ significantly in BAs and GABA in sourdough, bread crumb and crust prepared from Flour-2 chickpea. The differences were significant at the *p* < 0.05 level.

Baking had no effect on the content of BAs and GABA in the interior of the bread, whereas the content in the crust was significantly reduced (Figure 6), the magnitude of the effect depending on the type of BA. The largest relative decrease to about ⅓ of the content in the dough was observed for SPM and SPD, which was followed by PUT, CAD, and TYR, where, similarly to GABA, a 25 % lower amount was observed. The conversion of BAs in a real food matrix is a complex process where both increase and decrease in content has been previously noted. In roasted coffee [73], all analyzed BA were accumulated and the corresponding increase was more pronounced for BA, formed by decarboxylation, than for SPM and SPD, where the increase could be due only to the release of molecules covalently bound to the matrix. In the presence of reducing sugars, the content of HIS [74] and TYR [75] was decreased by the Maillard reaction after thermal treatment already at pH 6. During bread baking, reactions leading to both increases and decreases are potentially relevant. A larger relative decrease in SPD and SPM in the crust could be due to the Maillard reaction and to evaporation (the boiling points of SPM and SPD are 130 °C and 129 °C, respectively). Other analyzed BAs have higher boiling points, fewer amino groups per molecule, and may be formed by decarboxylation, all of which can contribute to lower losses. Since the crust is only a small part of the bread, baking does not considerably reduce the BA and GABA content in the bread. Therefore, the optimization of sourdough fermentation in terms of lower formation of BAs or their removal by diamine oxidase are necessary if the goal is to produce sourdough breads with a low BA content. The use of sprouts with high diamine oxidase activity could be considered beneficial, because the sourdoughs with the highest content of nutritionally undesirable BA, were also the richest in GABA.

## 4. Conclusions

The origin of chickpea flour has a major influence on the profile and amount of BAs and GABA formed in spontaneously fermented sourdoughs. CAD, PUT, and TYR accumulated to the highest extent. The differences in the contents of certain BAs in the sourdoughs prepared from flours of different origins were large and even reached up to two orders of magnitude. The addition of the commercial starter cultures did not result in lower levels of BAs in all sourdoughs.

The levels of nutritionally desirable GABA (up to 320 mg/kg DW) and antioxidants in the sourdoughs increased compared to the flours, depending on the flour, type, and duration of fermentation. In inoculated fermentations, GABA content was higher after 48 h compared to 24 h for all flours.

The addition of fenugreek sprouts, which are a rich source of diamine oxidases, to the sourdough suspension (pH 4.7) resulted in partial (40–50%) degradation of CAD, PUT, and TYR, while increasing the pH of the suspension to 6.5 allowed the complete degradation of nutritionally undesirable BAs. In dough, degradation was less efficient during kneading and resting, as about 40% of CAD and 10% of PUT and TYR were oxidized. Baking bread had a limited effect on the stability of BAs and GABA, as the contents were reduced only in the crust and not in the crumb compared to the dough.

Since the fermentation of chickpea dough is associated with the production of nutritionally undesirable BAs, their control is important to ensure the safety and quality of the fermented products. At the same time, different strategies could be used to prevent or minimize their quantity.

## Data Availability

The data presented in this study are available on request from the corresponding author.

## Appendix A

**Table A1:** Slope of calibration curves for biogenic amines and GABA obtained after fluorimetric and spectrophotometric detection. The concentration of biogenic amines and GABA used for the calculation of the calibration curve corresponds to the values in 250 μL of the calibration solution (mixture of standards in 0.4 M HCl) used in derivatization experiments.

Slope of Calibration Curves										
	GABA	TRP	PEA	PUT	CAD	HIS	IS2	TYR	SPD	SPM
UV (mAU × min × L/mg)	7.6	16.2	17.9	38.9	35.1	28.2	29.4	30.5	33.7	29.1
FL (signal × min × L/mg)	/	229	245	540	594	/	552	88.8	535	561

**Table A2:** Influence of fermentation on the pH value in three chickpea sourdoughs from Flour-1, Flour-2 and Flour-3, prepared by spontaneous fermentation (S) and inoculated fermentation with commercially available starter (I) after 24 h and 48 h of fermentation at 30 °C.

	Flour	24 S	48 S	24 I	48 I
Flour-1	6.12	5.78	5.02	5.27	5.34
Flour-2	6.10	4.74	4.34	5.18	4.95
Flour-3	6.17	5.67	5.09	5.04	5.00
